# Detection, Identification and Size Distribution of Silver Nanoparticles (AgNPs) in Milk and Migration Study for Breast Milk Storage Bags

**DOI:** 10.3390/molecules27082539

**Published:** 2022-04-14

**Authors:** Bin Li, Sew Lay Chua, Dingyi Yu, Sheot Harn Chan, Angela Li

**Affiliations:** National Centre for Food Science, Singapore Food Agency, 10 Perahu Road, Singapore 718837, Singapore; li_bin@sfa.gov.sg (B.L.); chua_sew_lay@sfa.gov.sg (S.L.C.); chan_sheot_harn@sfa.gov.sg (S.H.C.); angela_li@sfa.gov.sg (A.L.)

**Keywords:** silver nanoparticles, milk, migration study, breast milk storage bags, AF4-ICP-MS, SP-ICP-MS

## Abstract

The engineered silver nanoparticles (AgNPs) have been widely used in various food contact materials (FCMs) based on their antibacterial properties. This widespread use of nanosilver has, however, increased the risk of exposure of AgNPs to human due to their migration from FCMs causing a potential hazard present in foods. Therefore, it is important to establish a reliable and practical method for the detection of AgNPs in food matrices to support risk assessment on AgNPs exposure. Taking the examples of milk and AgNPs-containing breast milk storage bags, this study established an approach for size characterization and quantification of AgNPs in milk and evaluated the relevant silver migration, based on enzymatic digestion and the analysis by asymmetric flow field–flow fractionation (AF4) hyphenated with inductively coupled plasma mass spectrometry (ICP-MS) and single particle inductively coupled plasma mass spectrometry (SP-ICP-MS). No migration of AgNPs was found from breast milk storage bags under various simulated storage conditions as well as extreme scenarios. The suitability and reliability of this method were also validated by the determination of multiple parameters, including accuracy, repeatability, limit of detection (LOD), limit of quantification (LOQ), and recovery, for AF4-ICP-MS and SP-ICP-MS, respectively, with good and overall acceptable evaluation results obtained for all. The established and validated approach was demonstrated to be suitable for the characterization and quantitation of AgNPs in milk as well as the analysis of their migration from breast milk storage bags.

## 1. Introduction

Nanoparticles (NPs) exhibit unique physicochemical properties and are used in many consumer products, such as foods, packaging materials, cosmetics, and medicines [1,2,3]. Silver nanoparticles (AgNPs) are estimated to be one of the nanomaterials with the highest degree of commercialization in a wide range of application fields owing to their antibacterial properties [4]. In the food industry, AgNPs are widely used in food packaging materials due to their antimicrobial properties. As such, AgNPs are often applied in the inner coatings of various food storage containers to increase the shelf life of the contained food items [5]. However, the health risks associated with the use of such products due to the potential ingestion of Ag ions or nanoparticles which have migrated into food and drinks are relatively unknown, presenting a knowledge gap which needs to be addressed.

In the European Union, the regulation (EU) No. 1169/2011 requires engineered nanomaterials used as food ingredients be detected and labeled [6]. The European Food Safety Authority (EFSA) previously indicated the upper limits of silver (Ag) migration from packaging materials as ‘not exceeding the group specific migration limit of 0.05 mg Ag/kg food’ [7,8]. These criteria implied that evaluations of Ag migration profiles were necessary to assure the required antimicrobial effectiveness while complying with the current legislation in the EU. Similarly, the United States Environmental Protection Agency (USEPA) has banned the sale of some nanosilver-containing plastic food containers manufactured in the US due to the lack of proper quality testing and inspection of AgNPs according to the regulations of the United States Food and Drug Administration (USFDA) to ensure consumer safety [5]. Meanwhile, it is worthy to note that the EFSA concluded recently [8] that the safety of the additive silver nanoparticles used in plastics as a biocide, based on their existing form as “embedded in the polymer” and the level of potential migration of silver in its soluble ionic form was much lower than the allowed restriction. However, multiple preconditions for safety of the incorporated nanosilver were also raised and highlighted in this scientific option by EFSA, such as the maximum use level of 0.025% *w*/*w* for nanosilver in the polymer, only non-polar plastics (e.g., polyolefins, polyesters, styrenics) are allowed as the food contact material, etc., indicating the necessity to explore an approach to monitor silver migration for more universal scenarios.

As previously reported, there have been attempts to quantify the total Ag released from various consumer products, including food contact materials [9,10,11,12]. The overall conclusion from the experiments performed was that consumer exposure was negligible, as the amounts leached from the food containers were found to be very low and in the magnitude of ng/cm^2^. However, most studies only indicated the total amount of migrated Ag but did not differentiate between ionic silver (Ag^+^) and nanosilver with a certain size distribution, even though the size information of AgNPs was a key aspect for toxicity evaluation and relevant regulation in the legislation.

Nanosilver-containing food container/packaging has been marketed in some countries for many years, e.g., AgNPs lined breast milk storage bags. Several studies on the migration of AgNPs from various food contact materials into different foods or food simulants have been published [13,14,15,16]. However, there has been a lack of reports about sensitive and specific detection of the migration of AgNPs as antibacterial components from AgNPs-containing breast milk storage bags into milk. Breastfeeding has an irreplaceable function for the growth, immunity, and cognitive development of infants [17]. Since AgNPs-lined breast milk storage bags are widely used nowadays, especially by working mothers, the risk resulting from the potential migration of AgNPs from these storage bags into the breast milk, as well as their exposure to human health, has been raised as a food safety concern [18]. As infants are more sensitive than adults to toxins, even a lower number of AgNPs may result in unexpected toxic effects [18,19]. Hence, it is very important to assess the migration of Ag in the form of nanoparticles from breast milk storage bags into milk.

Asymmetric flow field–flow fractionation hyphenated with inductively coupled plasma-mass spectrometry (AF4-ICP-MS) and single particle inductively coupled plasma-mass spectrometry (SP-ICP-MS) have both emerged as highly promising and universal techniques in the analysis of AgNPs. For analysis by AF4-ICP-MS, fractionation relies on the physical separation of AgNPs over an ultrafiltration membrane under the effect of the cross-flow separation field, without involving any stationary phase. This feature makes AF4 particularly suitable for the size separation of AgNPs in complex mixtures. As shown in previous reports, AF4-ICP-MS has been applied to AgNPs in different food categories for its characterization, such as nutraceuticals and beverages [20], as well as complex matrices, such as chicken meat [21], with the reliable and reproducible determination of AgNPs’ mass fraction and size. Additionally, migration of nanosilver from food containers into simulants has also been explored by AF4-ICP-MS [22], with the significant finding that Ag had a low tendency to migrate from containers into foods from regular use. These studies demonstrated the advantages and limitations of AF4-ICP-MS in AgNPs characterization. In one aspect, AF4 can detect very small particles over a wide size range and has element-specific capabilities when coupled to ICP-MS. Even very small nanoparticles (e.g., 5–10 nm) can be well separated and detected by the AF4-ICP-MS, whereas the size detection limit of AgNPs by SP-ICP-MS is practically more than 20 nm. The ability of AF4-ICP-MS to detect small size changes allows one to determine the extent of complexation and aggregation of AgNPs clusters in a variety of matrices. However, the lengthy method development process and extra effort in the application of AF4-ICP-MS may be a drawback for some laboratories. With the hyphenation of AF4 to ICP-MS, the detection limit of ICP-MS may not be improved compared to other detectors such as UV or Dynamic Light Scattering (DLS) as there is dilution that takes place within the AF4 channel.

The SP-ICP-MS technique allows simultaneous counting and sizing of nanoparticles present in diluted aqueous samples with high sensitivity, i.e., at very low concentrations of sub-µg/L or even ng/L, which is the main advantage of SP-ICP-MS over other methods. Practically, SP-ICP-MS requires little sample preparation and additional method development for certain matrices, which is more important for the routine analysis of AgNPs. This technique had also been successfully applied to sizing and quantitative determination of AgNPs in various foods or human healthcare products, e.g., chicken meat [23], fruit juices [24], and consumer products [25], with good analytical performance assessed and validated. SP-ICP-MS also gives simultaneous distinction between dissolved and nanoparticulate constituents and can provide better resolution than many of the currently available instruments for sizing nanoparticles. However, it is highly dependent on the signal to noise ratio of a given ICP-MS system, which may significantly hinder the analysis of smaller sized AgNPs. Based on the advantages and limitations of these two techniques, the combination of AF4-ICP-MS and SP-ICP-MS in analysis of AgNPs may reveal comprehensive information for the reliable detection and characterization of AgNPs in foodstuffs in complex matrices.

Although the migration of AgNPs from commercial food contact materials (FCMs) has been studied over the past several years, mainly by SP-ICP-MS [23,24,25,26] and electron microscopy [27], the migration behavior of AgNPs from breast milk storage bags into milk has yet to be reported. In contrast, there have been many journal articles describing the characterization of AgNPs using AF4 coupled to various detectors such as Multi-Angle Laser Light Scattering (MALLS) or ICP-MS [20,21,28,29,30,31] due to AF4′s favorable nanoparticle size fractionation capability in complex samples. Hence, as far as we know, the application of AF4-ICP-MS for the detection and characterization of AgNPs in milk is the first to be undertaken by this study. In addition, SP-ICP-MS was also included herein on the consideration that this technique is considerably more sensitive than AF4-ICP-MS and offers the unique ability to simultaneously determine the concentrations of dissolved and nanoparticulate fractions of total Ag as well as the sizes of AgNPs [23,24,32]. Therefore, these two complementary techniques were utilized for analysis of AgNPs in milk.

In this study, we aim to (1) establish a validated method using AF4-ICP-MS and SP-ICP-MS systems for AgNPs identification, quantification, and size distribution in milk and (2) examine the migration of AgNPs from commercial breast milk storage bags. To the best of our knowledge, there has been no report on the detection and characterization of AgNPs in milk using the proposed approach, based on the combination and application of these two powerful techniques.

## 2. Results and Discussion

**Figure 1:**
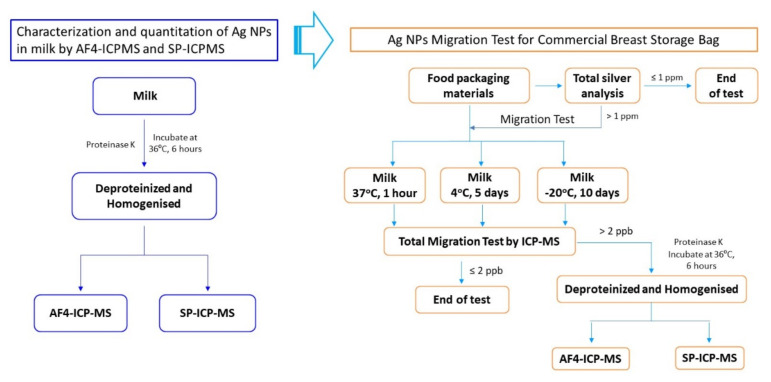
Experimental design for the characterization and quantitation of AgNPs in milk by AF4-ICP-MS and SP-ICP-MS, as well as the testing of AgNPs migrated from commercial breast milk storage bag into milk.

The experimental design shown in Figure 1 demonstrates the workflow in the current work for the characterization and quantitation of AgNPs in milk by AF4-ICP-MS and SP-ICP-MS, as well as the migration studies on AgNPs from commercial breast milk storage bags into milk.

### 2.1. Optimization for Extraction of AgNPs in Milk by Enzymatic Digestion

Matrices (simulants or milk) spiked with AgNPs standards were used throughout this study for method development and validation. Due to the potential interfering effects caused by the extensive presence of various matrix components in milk, especially proteins, various methods for the extraction of AgNPs spiked in milk with and without enzymatic digestion were explored and compared for their effective detection by AF4-ICP-MS.

**Figure 2:**
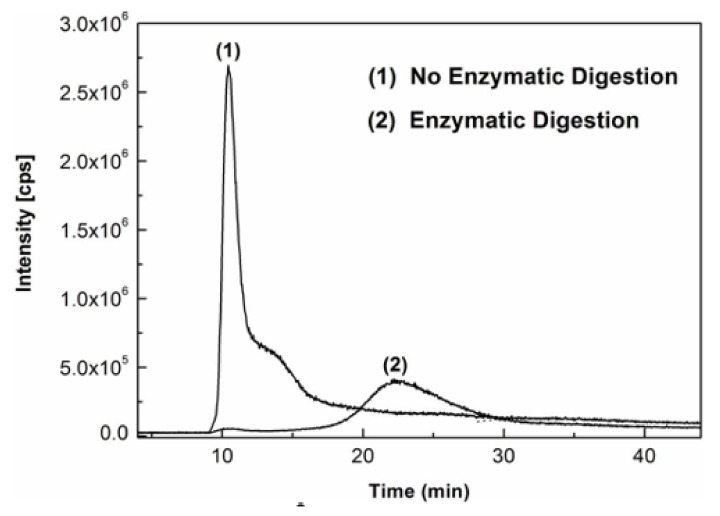
Overlaid AF4-ICP-MS fractograms of AgNPs standard (60 nm) spiked into milk (1) without or (2) with enzymatic digestion.

Figure 2 shows the overlaid AF4-ICP-MS fractograms of AgNPs standard of 60 nm diameter spiked into milk samples without or with enzymatic digestion. It was demonstrated that, when no enzymatic digestion was applied, most of the species of Ag were eluted in the void peak around 10–11 min, and small portion of Ag was eluted out as a shoulder peak at around 14 min without baseline AF4 separation from the void peak. In contrast, after enzymatic digestion of the milk sample, there was no obvious elution peak in the retention time range of 10 to 15 min and a well fractionated AF4 peak of AgNPs was instead detected around 22 min. As reported in previous papers [33,34], this phenomenon has been observed and well explained. In samples with high protein contents in the matrix, most of the proteins were unfractionated in the void peak after injection due to the incomplete hydrodynamic relaxation for large particles such as proteins in these scenarios. Meanwhile, for milk samples spiked with 60 nm AgNPs standard in this study, species of Ag mainly present as AgNPs can easily be bound to large particles of intact proteins in the solution, aided by several forces, such as hydrogen bonds, solvation forces, Van der Waals interactions, etc. Hence, most of the AgNPs bonded to proteins in milk were unretained and eluted in the void peak when there was no enzymatic digestion. However, when the milk was enzymatically digested, most of the milk proteins were degraded and a large number of AgNPs can be effectively retained on the AF4 membrane and well fractionated, as indicated by a well-defined AF4 peak at later time point. Similar to findings in previous reports [21,35], all results indicated that enzymatic digestion of proteins was necessary for proper AF4 separation and analysis of AgNPs in milk samples by AF4-ICP-MS in this study. Additionally, the results of size and number concentration measured by SP-ICP-MS analysis on the above enzymatic digest samples agreed well with expected values of the spiked 60 nm size AgNPs standard in aqueous solutions. As reported by other researchers [36], this observation also confirmed that the enzymatic sample preparation did not affect the particles in terms of size or agglomeration state.

Hence, enzymatic digestion was adopted for sample preparation in the following AF4-ICP-MS and SP-ICP-MS analysis.

### 2.2. Detection and Size Characterization of AgNPs in Milk by AF4-ICP-MS

As reported previously [37], the use of nanoparticle standards showed more advantages in size measurement since the same carrier composition and separation conditions in AF4 were applied for analytes of the same nanoparticles in both standards and samples. In addition, the AF4-ICP-MS technique normally had an advantage over SP-ICP-MS for effective analysis of small-sized nanoparticles by modifying the cross flow. In general, a higher cross flow rate was applied for the separation of smaller sized particles, while a lower cross-flow rate was used when larger particle sizes were anticipated. Hence, AgNPs standards (size-ladder) and AF4-ICP-MS analysis based on an optimized cross flow gradient program starting from 1 mL/min with linear decrement were both applied in current works.

To perform size calibration, the hydrodynamic sizes of the commercial AgNPs standard suspensions from the manufacturer’s certificates and their size distribution were first verified. As shown in Table 1, all the AgNPs size standards were analyzed by DLS and their hydrodynamic sizes in the form of Z-average were obtained. From the DLS analysis of each standard, the measured sizes agreed well with those declared by the manufacturer with the relative deviations in the range of 0.9 to 2.7% except for the 20 nm sized particles that showed RSD values of 6.0%. Meanwhile, it was also found that their particle size distributions were unimodal by DLS analysis, with the measured polydispersity indexes (PDI) ranging 0.077 to 0.244. All the results suggested that these commercial AgNPs standards presented highly monodispersed hydrodynamic sizes. Size calibration was generally completed in this study by singularly injecting AgNPs standard dispersions and plotting the hydrodynamic diameters reported in the manufacturer’s certificates against the resultant elution times of their peak maxima to obtain the regression curve for size calibration.

**Figure 3:**
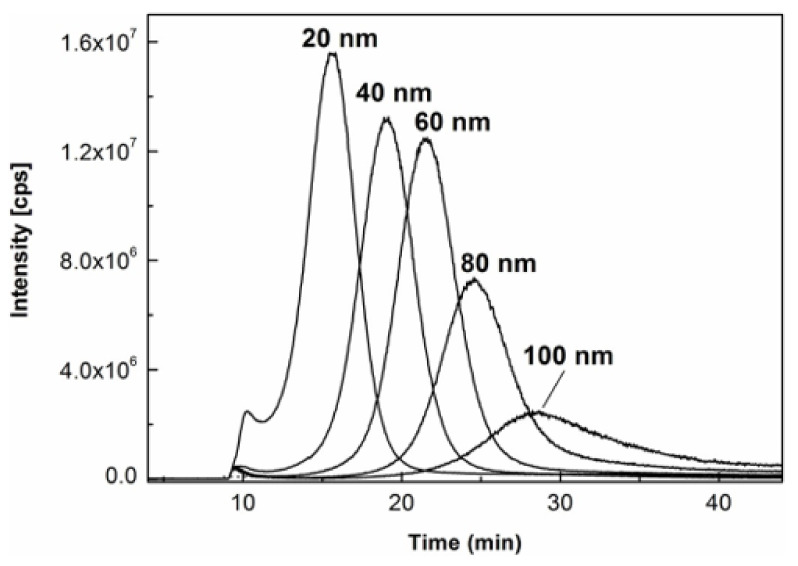
Overlaid AF4-ICP-MS fractograms of AgNPs standards dispersions in citrate buffer at 20 mg/L.

Figure 3 shows the overlaid AF4-ICP-MS fractograms of five AgNPs standards dispersed in citrate buffer at 20 mg/L with nominal sizes ranging from 20 to 100 nm. It was demonstrated that all peak maxima were separated and well-defined with successful fractionation. Nevertheless, the peak intensities of AgNPs standards of 80 and 100 nm obviously decreased and especially the fractogram peak of the 100 nm AgNPs standard became broadened over 20–40 min, indicating the possible loss and aggregation of the AgNPs standards due to their potential interactions with the AF4 channel under the matrix-free aqueous environment.

**Figure 4:**
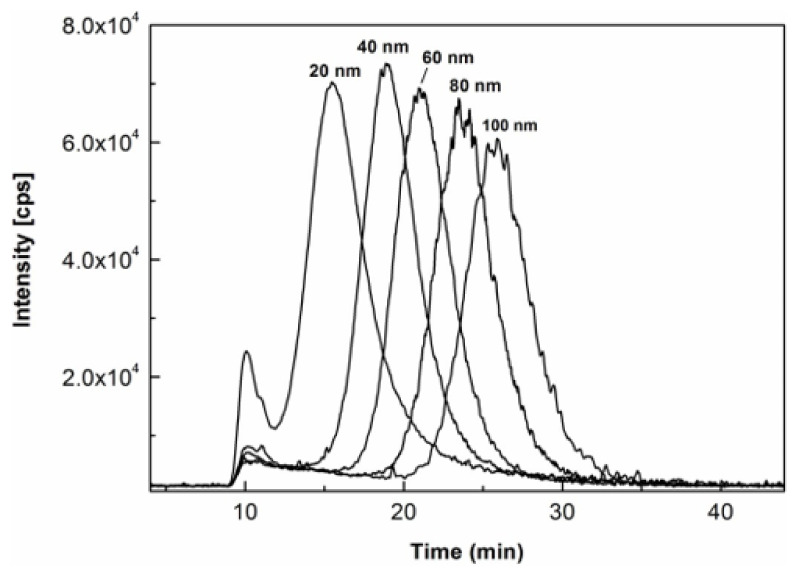
Overlaid AF4-ICP-MS fractograms of AgNPs standards (size-ladder) spiked into milk at 285 µg/L for each with enzymatic digestion at 36 °C for 6 h.

To further explore the effects of the milk matrix on size characterization of AgNPs, the same milk products spiked with the same AgNPs standards as those used in the analysis for Figure 2 were individually prepared with enzymatic digestion at 36 °C for 6 h and analyzed under the same AF4-ICP-MS conditions. As shown in Figure 4, their fractograms were generally displayed as symmetrical peaks with distinct peak maxima despite some of them being partially resolved, hence, allowing the attribution of AgNPs size in the unknown milk samples. The size calibration curve was then constructed by plotting the hydrodynamic diameters of the AgNPs standards declared by the manufacturer against their respective retention times of peak maxima. The modeling equation was determined to be y = 0.2367x^2^ − 2.4326x + 5.7161 (y is particle diameter in nm, x is Rt in min, R^2^ = 0.9998). The excellent polynomial fit between retention times and hydrodynamic sizes was theoretically anticipated based on the fact that there are generally parabolic flow velocity profiles inside the AF4’s channel [38]. It agreed with observations in previous studies utilizing AF4 [28].

A close comparison between Figure 3 and Figure 4 revealed that the AgNPs standard of 100 nm was present as symmetrical and broad peak in citrate buffer while being relatively symmetrical and narrow peak in milk matrix. To adopt the best suitable strategy for size characterization of AgNPs in the migration study, it was necessary to compare and rationalize the different AF4 behaviors of AgNPs under these two circumstances.

Compared with AgNPs, other large particles in milk matrices (e.g., protein, peptide, lipid, etc.) had larger particle sizes and lower diffusion coefficients. As discussed in some previous reports [21,35], these types of big matrix particles were carried to the slower stream lines of the AF4 channel and pushed relatively smaller AgNPs out to the faster stream lines, making the AgNPs eluted earlier. The strength of this interference effect increased with the increment of the size of AgNPs since the diffusion coefficient decreased and the diffusion speed of AgNPs turned slower with the increment of their sizes, which led to a higher probability for the matrix components to interfere and interact with the larger AgNPs. Furthermore, the interactions between milk matrices and the AgNPs standards can also stabilize the primary AgNPs by reducing their electrostatic strength with the AF4 membrane and minimize the probability of aggregation. All these interferences could make the AgNP standards in Figure 4 eluted earlier than their counterparts in Figure 3, especially for the AgNP standards with bigger sizes of 80 and 100 nm. The difference in elution behavior of these two scenarios indicated that it is more suitable to use AgNPs standards spiked in milk for the construction of the calibration curve for size determination in milk as it better mimics the behavior of AgNPs in unknown milk samples.

### 2.3. Characterization and Determination of Number Concentration of AgNPs in Milk by SP-ICP-MS

For SP-ICP-MS analysis, samples containing metal or metal-oxide nanoparticles were nebulized directly when introduced into the ICP-MS system, resulting in a non-homogeneous distribution of the analyte in the aerosol droplets and a flash of gaseous ions in the plasma. Each single particle generated an ion plume which can be detected as a transient peak signal (in the range of about 300 to 500 μs) in the plasma and a subsequent spike in the graph when appropriate low dwell times for the time-resolved data acquisition were chosen. The sample had to be highly diluted with ultrapure water to achieve the targeted concentration approximately in the level of ng/l for SP-ICP-MS measurement, so that only one particle may reach the detector per chosen dwell time interval.

**Figure 5:**
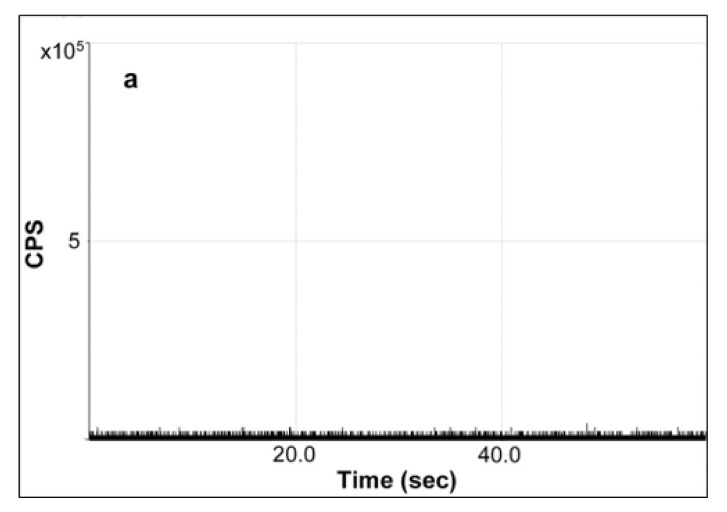

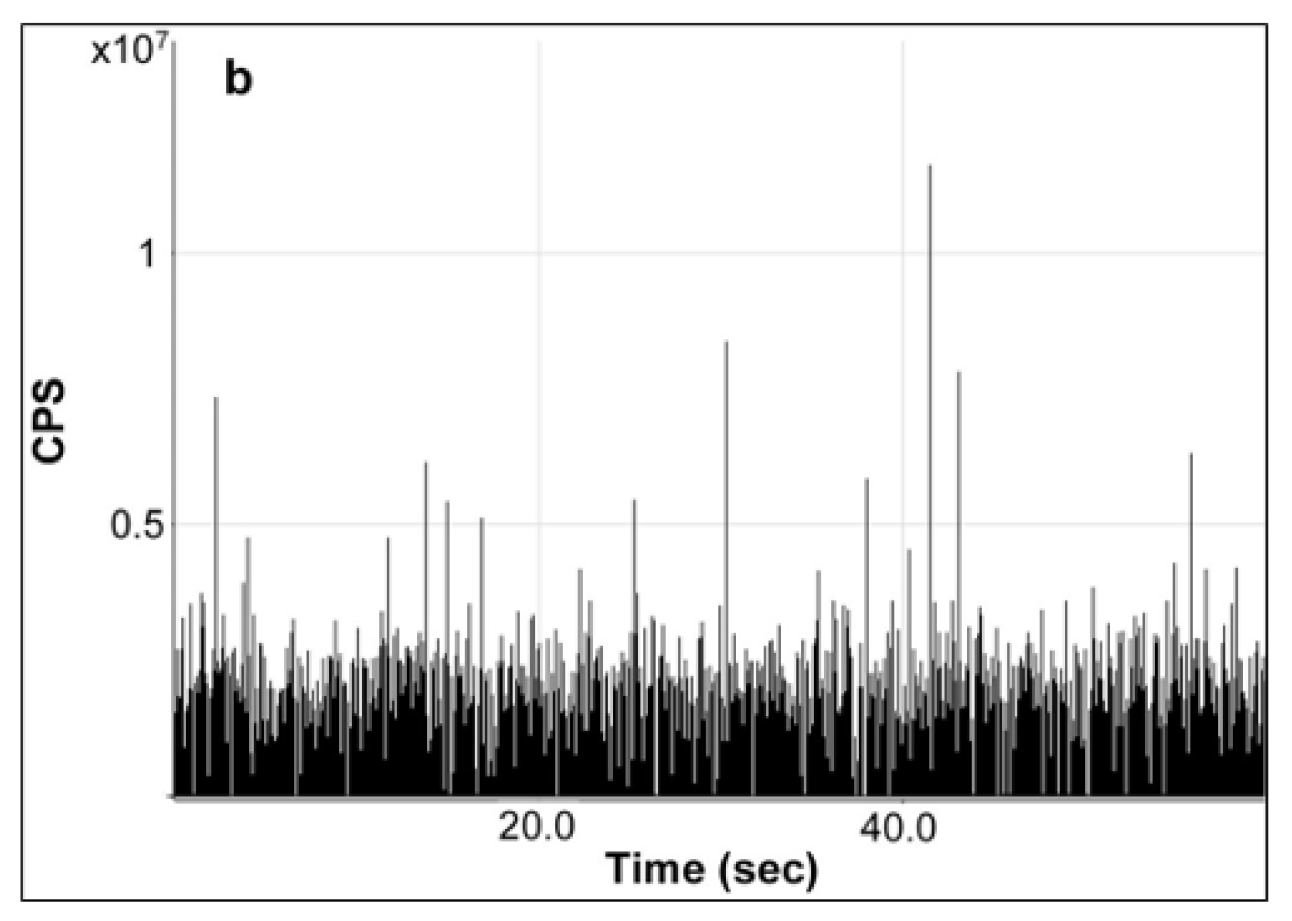
SP-ICP-MS time scan of ^107^Ag for analysis of samples of (**a**) the water blank and (**b**) the diluted enzymatic digest of milk spiked with 80-nm AgNPs standard at a mass concentration of 285 µg/L.

As shown in Figure 5a, there was no spike in the background signal obtained in the representative time scan of a water blank sample. Since the baseline of raw signal in the original time scan corresponded directly with the background amount of Ag^+^ which may interfere or even hinder the detection of AgNPs in sample, the absence of obvious high background signals of Ag enabled the reliable detection and determination of AgNPs, especially those migrating at a trace level. In comparison, the characteristic raw time scan of the diluted enzymatic digest of milk extract spiked with 80-nm AgNPs at a concentration of 285 µg/L showed significant spikes as observed in Figure 5b. Due to the very low Ag^+^ background and interference observed, a sufficient number of single particle events can be detected for this milk digest, clearly indicating the presence of AgNPs. It is also worth mentioning that although the detection of AgNPs in milk could be achieved without any sample preparation prior to analysis as in a previous report [24], the enzymatic digestion method was adopted for SP-ICP-MS analysis in alignment with the same treatment which was necessary for favorable fractionation in AF4-ICP-MS analysis, avoiding or minimizing the potential bias or discrepancy caused by different sample preparation protocols.

The quantification for the number concentration of AgNPs can also be performed via SP-ICP-MS analysis. By obtaining the signal distribution of the raw transient signal/spikes from the time scan, a particle detection limit can be determined, which in turn determined the number of detected nanoparticles. Based on the ionic calibration, particle mass and particle diameter can be calculated from the signal intensity of the detected spikes. Thus, the particle number concentration can be calculated from the number of detected particles in the measured time interval, the transport efficiency, and the sample input flow.

**Figure 6:**
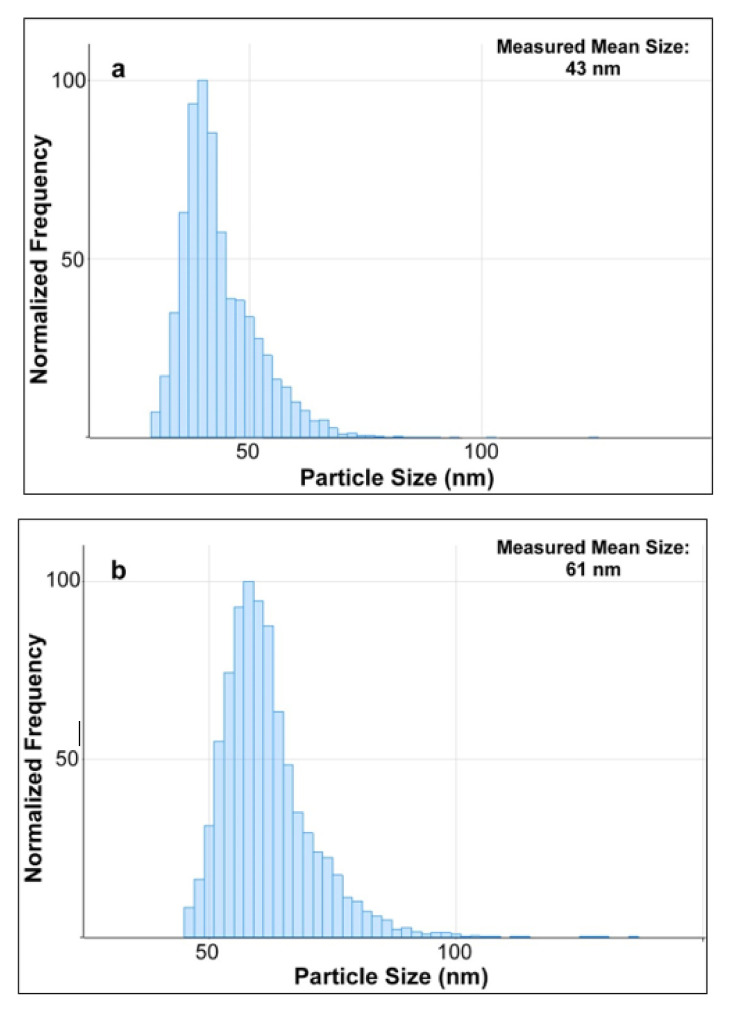

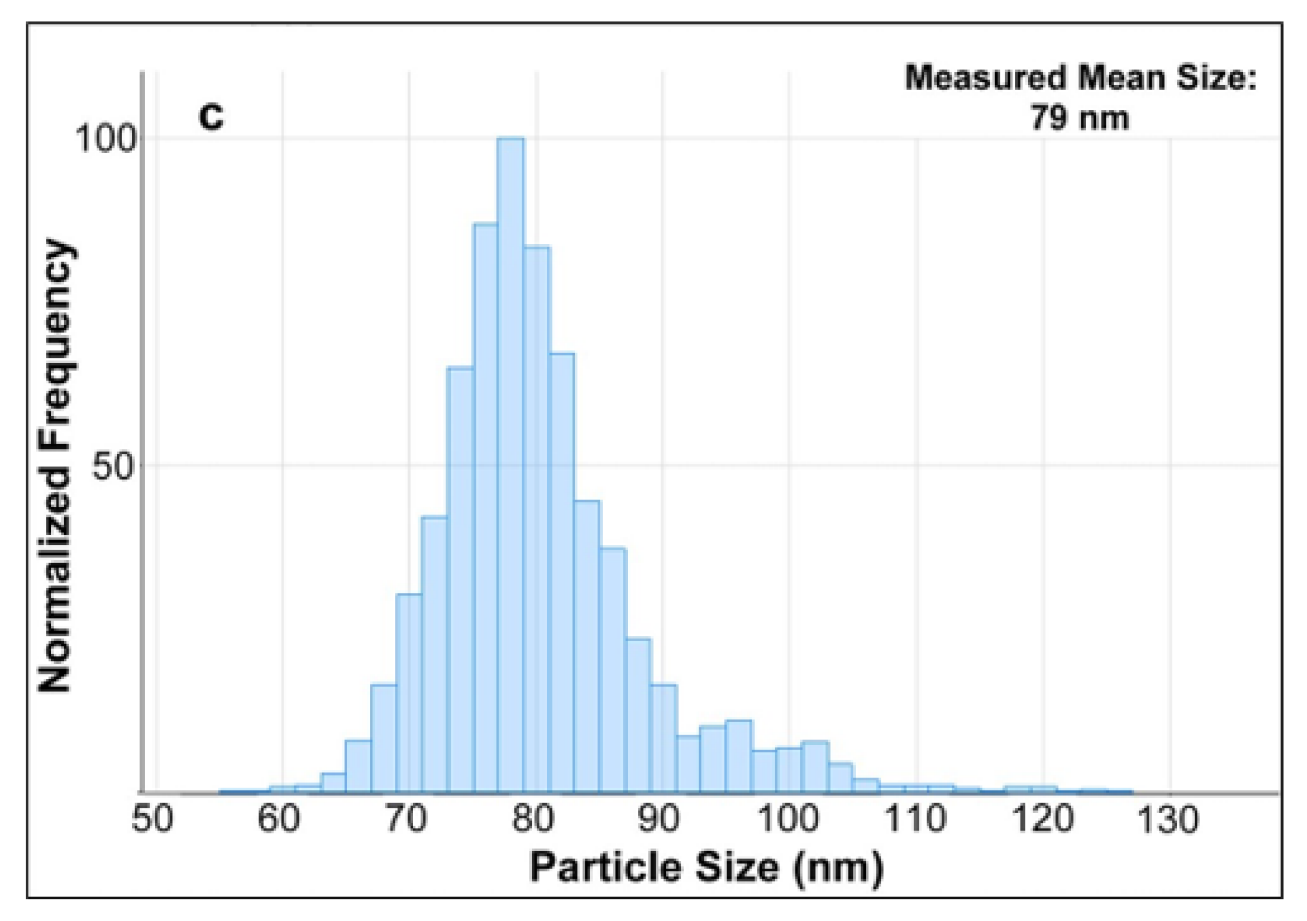
The experimental particles size distribution of AgNPs of (**a**) ~40 nm, (**b**) ~60 nm, and (**c**) ~80 nm spiked in the diluted enzymatic digest of milk, as measured by SP-ICP-MS analysis.

Three samples of the diluted enzymatic digest of milk spiked with AgNPs standards of 40, 60, and 80 nm were then measured by SP-ICP-MS. By using the MassHunter software which utilizes a series of algorithms to process the acquired SP-ICP-MS data, the particles size distribution of AgNPs were obtained and shown in Figure 6. Compared with the TEM diameters of 41, 59, and 81 nm (Table 1) of these three AgNPs standards claimed by the manufacturer, their corresponding measured mean sizes of 43, 61, and 79 nm slightly varied with a maximum shift of ±2 nm. Although the results were based on different analytical techniques, they were still in good agreement, which indicated that the TEM results further verified the characterization of AgNPs in milk by using SP-ICPMS in this study. On the other hand, number concentrations of AgNPs in these three spiked samples were measured by SP-ICP-MS as well, and they were all in good agreement with the values reported in the manufacturer’s certificates of AgNPs standards with a recovery of 97 ± 3% (n = 3). Considering the significantly low background signal of Ag in SP-ICP-MS analysis discussed as above, this approach was used to enable the detection and determination of AgNPs in their migration studies as discussed in later sections.

### 2.4. Validation and Evaluation of the Established Methods

Accuracy and repeatability—Repeated injections of milk samples spiked with AgNPs standards of various sizes and concentrations were evaluated in terms of accuracy and repeatability precision. Accuracy was determined by comparing the average measured AgNPs concentrations or sizes to their theoretical values, respectively. Repeatability was determined by the calculation of the relative standard deviation (RSD) of the measured concentrations or sizes of certain AgNPs. The results are shown in Table 2. It was found that favorable accuracies were obtained, ranging from 96 to 101% and 87 to 106% in the determination of concentration and size by AF4-ICP-MS, as well as 99–101% and 97–103% for the measurement of their counterparts by SP-ICP-MS. Since the accuracy was considered acceptable if they fell within 80–120%, the established methods of AF4-ICP-MS and SP-ICP-MS were therefore deemed to be adequately accurate for the analysis of AgNPs in milk. Moreover, all the measured RSD were 1 or 2% which was much less than the general RSD threshold of 5% for chemical analysis in ppm level. These observations further indicated the suitability of testing with the established methods.

LOD and LOQ—Since the detector response when using ICP-MS was mostly independent of the size of AgNPs [28], 60-nm AgNPs was typically utilized to determine the LOD and LOQ in the analyses by AF4-ICP-MS and SP-ICP-MS. Table 3 shows the detection and quantification limits of AgNPs in milk obtained for both AF4-ICP-MS and SP-ICP-MS. The LOD was found to be 3.2 µg/L for AF4-ICP-MS and 0.5 µg/L for SP-ICP-MS, and the LOQ was measured as 10.6 µg/L for AF4-ICP-MS and 5.6 µg/L for SP-ICP-MS, respectively. In general, LOD and LOQ of AgNPs determined in the current works were comparable with previous reports by other researchers for analysis of AgNPs in various types of samples, such as dietary food supplements [39], environmental samples [25,40], and aqueous suspensions with deionized water [41]. Differences among these studies can partly be attributed to differing injection volumes and/or differing instrumental performance characteristics and instrument set-ups, such as the type of nebulizer used.

Consistent with the previous report [33], AF4-ICP-MS in this study represented the lower detection sensitivity than that of SP-ICP-MS, with the LODs at levels of µg/L vs. sub-µg/L, respectively. However, referring to the reported LOD of AF4-ICP-MS at 6.7 µg/L shared by all AgNPs within the size range of 10 to 100 nm [28], the LOD of AF4-ICP-MS in the current work was reasonably expected to enable sensitive detection of AgNPs at least down to 10 nm at the lower end of µg/L (e.g., 3.2 µg/L or 3.2 ppb), because the detection response of ICP-MS was mostly independent of the size of AgNPs on a mass basis. Considering the maximum limit of Ag migration as 0.05 mg/kg (i.e., 50 ppb) in foods permitted by EFSA [7,8], it was implied that the AF4-ICP-MS could bear sufficient detection sensitivity in this migration study on AgNPs, with a detected size range wider than that by SP-ICP-MS.

Recovery Rate—The recovery of Ag NPs was an important aspect to take into consideration for the analysis of AgNPs in milk by AF4-ICP-MS and SP-ICP-MS. In this study, the recoveries of 60-nm AgNPs standard dispersions in milk by these two techniques were investigated. It was found that recoveries were favorable for both of the methods, ranging from 104 ± 10% (n = 3) for AF4-ICP-MS to 97 ± 7% (n = 3) for SP-ICP-MS, generally suggesting that there was no obvious loss of AgNPs coming from either extraction and interference by the milk matrix or the instrumental parameters (e.g., cross flow in AF4 separation or dwell time in SP-ICP-MS), and that the established two approaches are appropriate for the quantification of AgNPs in milk.

### 2.5. Determination of Total Silver Content in Breast Milk Storage Bags

Three kinds of breast milk storage bags labeled with the presence of nanosilver were first investigated in this study for method development. As shown in Table 4, the total Ag contents present in these bags ranged from 3.48 to 22.89 mg/kg, comparable with the detected total Ag content in breastmilk storage bag by ICP-MS which was reported previously (i.e., 24.80 ± 0.09 mg/kg after acid digestion) [42]. The recovery of total Ag determined with microwave-assisted digestion followed by ICP-MS analysis was 95 ± 3% (n = 3) in this work.

### 2.6. Migration Test of Breast Milk Storage Bags

According to the European Regulation 10/2011 [43], for the evaluation on migration of materials and articles, testing for 10 days at 40 °C shall cover all storage scenarios in refrigerated and frozen conditions which are applicable to packaging and storage of commercial milk products. Hence, the simulant of 10% ethanol was utilized for studies on migration of AgNPs in milk in this work. Different incubation conditions were herein applied for migration studies to determine the content of total silver (Ag^+^ and AgNPs) migrated into 10% ethanol simulant and milk, as shown in Table 4.

The concentrations of total silver (Ag^+^ and AgNPs) migrated from the three investigated breast milk storage bags into the food simulant of 10% ethanol after 10 days of exposure at 40 °C are shown in Table 4. It was found that total amounts of Ag migrated, ranging from 0.16 to 0.28 µg/L, were consistent with the ones in previous studies claiming low migration of Ag measured at µg/L level [42,44].

Migration of total Ag from these investigated breast milk storage bags into milk was then explored. Firstly, the LOD of total Ag in milk was determined to be 0.27 µg/L (n = 3), based on microwave-assisted acid digestion and measurement by ICP-MS. This LOD value was comparable with those obtained in the migration test by using 10% ethanol in this study, suggesting the mild interfering effects from milk matrix and the suitability of 10% ethanol as a simulant in the migration study of AgNPs in milk. Subsequently, content determination of total Ag migrated into milk was conducted with the three tested breast milk storage bags. All the measured values were below the LOD of 0.27 µg/L, considered as the background noise and no detection of the total Ag migrated into milk. To validate this established approach for testing total Ag migrated into milk, an additional six commercial breast milk storage bags were further involved to conduct a surveillance study with larger sample size. Once more, there was no detection of total Ag migrated into milk for all these surveillance samples, indicating the absence of silver migration from all the investigated breast milk storage bags into milk under the migration conditions in this work.

Moreover, various extreme incubation conditions were further applied to explore the potential migration of Ag under extreme conditions from the storage containers into milk, including heating at 70 °C for 1 h, boiling at 100 °C for ½ h, and microwaving for 1 min. Once again, the obtained results showed that there was no migration of Ag. Therefore, there is evidence to infer that the AgNPs were completely embedded by polymers in the milk storage containers and the contact surface in these containers would not be altered by mechanical surface stress under various migration testing conditions, even after the harsh conditions.

In consideration of no detection of silver migration from all the investigated breast milk storage bags into milk, features of the established approach can then be summarized. First, with a series of AgNPs standards (size-ladder) applied as representative mimic analytes of the migrated AgNPs, the important parameters, such as size/size distribution, mass and number concentration, elemental compositions, etc., can be well characterized with the combination of multiple techniques in this work. Consequently, even there is generally no specification information provided about the incorporated nanosilver, except the labeled claim of “nanosilver”, for almost all commercially available AgNPs-containing milk storage bags and other similar products, the main profiling of the potentially migrated AgNPs is expected to be characterized with the established method in this work. Second, the generally low abundance of the incorporated nanosilver in the containers makes their detection more difficult, especially for the monitoring of AgNPs migration into complex food matrices (e.g., milk). Therefore, the alternative mimic analytes of the AgNPs standards had to be used for the validation and evaluation of the established methods instead of the actually migrated AgNPs. It is worth noting that there was a low migration of total silver (Ag+ and AgNPs) positively detected by using of 10% ethanol. Due to the absence of the interfering effects from the food matrix, this food simulant represents its complementary value for the studies on AgNPs migration into foodstuffs.

## 3. Materials and Methods

### 3.1. Chemicals and Materials

The mixed surfactant NovaChem Surfactant 100 (NC) comprising nonionic and ionic detergents was obtained from Postnova Analytics (Landsberg am Lech, Germany). Proteinase K from Sigma-Aldrich (Saint Louis, MO, USA) was utilized in a digestion buffer consisting of 10 mM Tris-HCl (pH 7.5) and 1% (*w*/*v*) Triton X-100, which were both from Sigma-Aldrich. Hydrogen peroxide (H_2_O_2_) (30%, UltraPure Reagent) and plasma-pure nitric acid (HNO_3_) (67–69%) were obtained from J.T. Baker (Center Valley, PA, USA). The standard solutions of Silver (Ag) (1000 µg/mL) and Rhodium (Rh) (1000 µg/mL) were obtained from Reagecon Diagnostics Ltd. (Shannon, Ireland) and used for ICP-MS testing. Ethanol (ACS reagent grade) was purchased from Merck (Darmstadt, Germany). A working concentration of 10% (*v*/*v*) ethanol was freshly prepared via dilution with water. Milli-Q ultrapure grade water was used throughout the work.

AgNPs dispersions (NanoXact) with 20, 40, 60, 80, and 100 nm nominal diameters were purchased from nanoComposix (San Diego, CA, USA). All standards were supplied as suspensions of AgNPs at the mass concentration of 20 mg/L and stabilized in a citrate buffer. In-house characterization of these standards before use proved the accuracy of the number concentrations of AgNPs and total Ag contents declared by the manufacturer, suggesting the sufficient reliability of their application in this study. Additionally, suspension of gold nanoparticles (AuNPs) with a nominal size of 40 nm (54 mg/L) from nanoComposix was used to detect nebulizer transport efficiency and nebulization efficiency.

The commercial fresh milk product was homogenized and pasteurized skimmed milk containing less than 1% fat content, purchased from a local supermarket and used for this study. In total, nine kinds of commercial breast milk storage bags labeled with the presence of nanosilver were purchased on-line and used in this study. Their product information is summarized below. (1) Three kinds of bags were used for method development, including two being low-density polyethylene (LDPE) bags (Perfection Premium a Grade Mother’s Milk Pack (JACO, Seongnam-si, Gyeonggi-do, Korea) and a Breast Milk Storage Bag (Zhejiang Rikang Baby Products, Taizhou, China)), and one as a multilayer PET/PE bag (Perfection Nano Silver Breast Milk Storage Bag (JACO, Seongnam-si, Gyeonggi-do, Korea); (2) Six kinds of bags used for the surveillance study, including two being low density polyethylene (LDPE) bags (Breast Milk Storage Bag (Phyll (Hansung Company), Seoul, Korea) and Breast Milk Storage Bags (Nevi, Seoul, Korea)), and four being multilayer PET/PE bags (Nanosilver Breast Milk Storage Bag (Ubee, Seongnam-si, Gyeonggi-do, Korea), a Perfection Premium Grade Breast Milk Storage Bag (JACO, Seongnam-si, Gyeonggi-do, Korea), a Nano Silver Breast Milk Storage Bag (Snow Bear, Pocheon, Gyeonggi-do, Korea), and a Breast Milk Storage Bag (Shi Yue Jie Jing, Xiamen, China)).

### 3.2. Instrumentation

An Agilent 7900 ICP-MS equipped with a Micromist nebulizer and a Scott type spray chamber (Santa Clara, CA, USA) was used for all ICP-MS, AF4-ICP-MS, and SP-ICP-MS experiments. Instrument tuning was performed prior to analysis to obtain a high sensitivity and minimize the presence of oxide and doubly charged species. Instrument parameters were set as follows: RF power, 1550 W; Plasma gas (Ar), 15.0 L/min; Carries gas (Ar), 1.09 L/min; Auxiliary gas (Ar), 0.9 L/min. The ICP-MS system was operated in He mode and the isotope of *m*/*z* 107 for Ag was monitored with an integration time of 0.5 s. The internal standard of Rh at *m*/*z* 103 was used for correction in total Ag determination by ICP-MS.

Prior to the determination of the elemental concentration of total Ag in the forms of ionic species and nanoparticles, ICP-MS was calibrated using a blank control and five Ag standard solutions. One of the two calibration ranges was used for the determination of total Ag content after digestion in the migration studies, i.e., 10, 20, 50, 100, and 500 µg/L or 0.2, 0.5, 1.0, 2.0, and 5.0 µg/L, depending on the predicted level of Ag concentration.

A Zetasizer Nano ZS from Malvern Instruments (Malvern, UK), maintained at 1.330 and 0.8872 cp for refractive index and viscosity respectively so as to mimic the corresponding parameters of pure water, was utilized to perform the DLS measurement in the evaluation of size distribution of AgNPs (based on hydrodynamic diameters).

For AgNPs analysis by AF4-ICP-MS, an AF2000 system from Postnova Analytics (Landsberg am Lech, Germany) was coupled to the Agilent 7900 ICP-MS. The AF4 system consisted of a regenerated cellulose membrane with a molecular weight cut-off of 10 kDa and a spacer thickness of 350 μm. AgNPs separation was achieved by using 0.1% NovaChem 100 as the AF4 carrier. A constant cross flow of 1 mL/min was applied initially, while the flow rate to the detector was kept constant at 0.5 mL/min. After a period of 7 min for sample injection and 1 min for focusing and transitioning, the cross flow was kept constant for 0.2 min and decreased linearly to 0 mL/min over 35 min. The tip flow was decreased as well until a flow rate of 0.5 mL/min was reached and kept constant for 10 min, yielding a total AF4 run time of 53.3 min.

The determination of AgNPs by SP-ICP-MS was also conducted by the Agilent 7900 ICP-MS system in the single particle mode, with the isotope of *m*/*z* 107 for Ag monitored and measured. The 0.1 ms dwell/integration time was used to minimize the probability of particles overlapping in the integration window. The duration of acquisition/measurement time was typically 60 s. In general, for routine SP-ICP-MS analysis, an Ag^+^ solution at a concentration of 10 µg/L was analyzed for the instrument calibration and a 40 nm gold nanoparticles standard from nanoComposix (San Diego, CA, USA) was measured to derive the nebulization efficiency of ICP-MS. The AgNPs standard suspension (60 nm) was freshly diluted to a concentration of 20 ng/L and measured by SP-ICP-MS to simultaneously calibrate and assure the instrument performance. After each data acquisition, the software automatically integrated the peak area of each single particle and generated information about particle size distribution.

### 3.3. Sample Preparation of AgNPs in Milk

Based on the modification of methods established previously [23,45,46], the enzymatic digestion was applied in this study. In brief, enzymatic digestion of the sample was carried out in two steps. First, 2 mL of digestion buffer was added to 5 g of sample and the mixture was vortexed for 1 min. Second, 25 µL of proteinase K (22 mg protein/mL) was added to the mixture and vortexed for 30 s. The tubes were subsequently incubated for 6 h at 36 °C. Milk was spiked with the AgNPs standard (60 nm) for AF4-ICP-MS and SP-ICP-MS analysis at concentrations of 20, 40, 60, 80, 100 µg/L and 2, 4, 6, 8, 10 µg/L, respectively. After enzymatic digestion with Proteinase K, the spiked milk samples were cooled to room temperature and homogenized. The extracts were individually injected into the AF4-ICP-MS for analysis, while each of the same extracts had to be diluted with water 100-fold before their SP-ICP-MS analysis.

### 3.4. Method Characterization

To evaluate and verify the method reliability, the established methods of AF4-ICP-MS and SP-ICP-MS were subjected to the detailed characterization of their analytical performance.

#### 3.4.1. Accuracy and Repeatability

Accuracy was defined as the closeness of agreement between a test result and the accepted reference value. The accuracies of the measured size and concentration of AgNPs were assessed by spiking AgNPs standards into sample matrices at five different concentrations ranging from 20 to 100 µg/L and with sizes in the range of 20 to 100 nm, respectively, over three different days. Repeatability was defined as the closeness of the agreement between repeatedly injected test samples. Generally, repeatability for the determination of Ag concentration and size was expressed as the relative standard deviation (RSD) based on the observed concentration or size of AgNPs. To assess repeatability for the mass concentrations analysis by AF-ICP-MS and SP-ICP-MS, six milk samples spiked with 60-nm AgNPs at a concentration of 60 µg/L and 6 µg/L, respectively, were prepared individually for 3 sets and analyzed over three different days. To assess repeatability for the AgNPs size measurement, the same procedures as above were repeated for analysis by AF4-ICP-MS and SP-ICP-MS, with the only exception that the AgNPs-spiked milk samples were prepared at a spiking concentration of 285 µg/L. The measured concentrations and sizes were then compared with the theoretically expected values to calculate accuracy and repeatability.

#### 3.4.2. Limit of Detection/Quantification

To determine the limit of detection and quantification, 60-nm AgNPs was selected as a representative example and a series of its standard dispersions with decreasing concentrations were injected for analysis by AF4-ICP-MS and SP-ICP-MS. The limit of detection (LOD) was determined as the lowest concentration of the AgNPs in milk sample that can be detected. It was calculated based on the formula: LOD = 3 × (residual standard deviation of regression line/slope of calibration curve). The limit of quantitation (LOQ) was defined as the lowest concentration of the AgNPs in the milk sample that can be determined with acceptable precision and accuracy under the operational conditions of the methods. It was calculated based on the formula: LOQ = 10 × (residual standard deviation of regression line/slope of calibration curve). In these formulae, the residual standard deviation of the regression line and slope of the calibration curve were derived from the plot of the instrument response versus the concentration of spiked milk samples. The determination was performed with triplicate across three different days, and average reading was taken as the LOD or LOQ for the analysis.

#### 3.4.3. Recovery Rate

The recovery rates of AgNPs for measurement by AF4-ICP-MS and SP-ICP-MS were investigated as well, exemplified by the analysis of milk samples spiked with 60-nm AgNPs at the concentrations of 285 µg/L for AF4-ICP-MS and 285 µg/L for SP-ICP-MS, respectively. In short, the recovery rates were determined by the comparison of the analyte signal in the pre-spiked (blank) milk to that in the post-spiked milk sample, i.e., the area counts of AgNPs peak in fractogram by AF4-ICP-MS or the number concentrations of AgNPs detected by SP-ICP-MS.

#### 3.4.4. Determination of Total Silver Content in the Materials

For the three commercial breast milk storage bags used for method development in the migration study, each was cut into small pieces, respectively, and about 10 mg of these plastic fragments were weighed out into PTFE digestion vessels and digested in a microwave reaction system (CEMS6, CEM Corporation, Matthews, NC, USA) with 2 mL of H_2_O_2_ and 10 mL of HNO_3_. Digestion was performed by increasing the temperature from 25 to 200 °C over 30 min then maintained for 15 min. After cooling the vessels, each digested sample was diluted with water and analyzed by ICP-MS for total Ag content.

ICP-MS was also utilized for the determination of total Ag content in the simulants/incubated milk samples for migration assay. After incubation, the simulants were sonicated for 5 min and cooled to room temperature. Ten milliliters of each migration extract was then transferred into a metal-free 15 mL tube and acidified with 0.2 mL of HNO_3_. Milk samples incubated after storage in −20, 4 and 37 °C conditions were also individually analyzed, in which samples treated under −20 and 4 °C were further placed in a 37 °C water bath for 30 min before the subsequent sample preparation. Two milliliters of H_2_O_2_ and 10 mL HNO_3_ were added into 0.8 mL of the treated milk samples for digestion by microwave. After cooling to room temperature, the digest was diluted to a total volume of 50 mL with water and homogenized prior to injection for ICP-MS analysis.

Furthermore, the LOD of total Ag in milk was explored. A linear regression for response versus concentration of spiked milk samples was plotted at five spiking levels. The resulting gradient and standard deviation of regression, obtained using Excel’s LINEST function, was used to derive the LOD of total Ag in milk. The ionic Ag standards at concentrations of 0.5, 1.0, 2.5, 5.0, and 7.5 µg/mL were spiked in 0.8 g milk samples and digested in a microwave reaction system (CEMS6, CEM Corporation, Matthews, NC, USA) with the addition of 2 mL of H_2_O_2_ and 10 mL of plasma-pure acids of HNO_3_ (67–69%), followed by dilution with water and analysis by ICP-MS to determine total Ag content. The measurement based on the above whole process was repeated thrice over three different days.

#### 3.4.5. Migration Studies

The migration testing was carried out according to the European Regulations on plastic materials and articles for the purpose of food contact (European Commission, 10/2011/EU) [43]. In our experiments, the migration of AgNPs was evaluated using 10% ethanol as a simulant, as well as the actual food matrix of milk. Three kinds of breast milk storage bags for method development in migration study were filled with 250 mL of simulant solution or milk, respectively, for incubation under different temperatures maintained in the corresponding equipment, including freezer, refrigerator, and oven.

A surveillance of an additional six kinds of commercial breast milk storage bags was subsequently carried out to determine the content of total Ag migrated into milk at 37 °C for 1 h, 4 °C for 5 days, and −20 °C for 10 days.

## 4. Conclusions

An effective method for the characterization and quantification of nanosilver in milk and relevant migration test of AgNPs using AF4-ICP-MS and SP-ICP-MS was developed and validated. Based on the optimized extraction of AgNPs in milk by enzymatic digestion, the established sample preparation method enabled the compatible and effective analysis of AgNPs by AF4-ICP-MS and SP-ICP-MS. The on-line AF4-ICP-MS analysis was accomplished for accurate dimensional characterization of AgNPs in the milk extract with size calibrants. The size characterization and quantification (e.g., particle mass and number concentrations) of AgNPs in milk were conducted by SP-ICP-MS as well, with reliable and accurate detection achieved, such as a maximum shift of ±2 nm in size measurement of AgNPs and a recovery of 97 ± 3% in testing number concentrations of AgNPs. The favorable testing capabilities of the method to generate accurate, reproducible, and sensitive results was also verified by the determination of multiple parameters in AF4-ICP-MS and SP-ICP-MS analysis, such as accuracy, repeatability, LOD, LOQ, as well as recovery rate. Among them, the LODs determined as 3.2 µg/L for AF4-ICP-MS and 0.5 µg/L for SP-ICP-MS were proved to be comparable with many previous reports, enabling the sensitive and reliable detection of the migrated AgNPs at trace level. This study showed the absence of Ag migration from commercially available breast milk storage bags (with the incorporated nanosilver claimed) into milk under various simulated migration conditions.

The combination of AF4-ICP-MS and SP-ICP-MS is a promising and complementary analytical platform for the detection and quantification of AgNPs in milk, as well as the monitoring of any possible release of nanosilver from FCMs into milk. It is expected that the developed analytical methodology could also be applicable to other similar dairy-related foodstuffs containing AgNPs with appropriate modifications for nanosilver-related safety assessment studies.

## Figures and Tables

**Table 1:** Evaluation of the distribution of commercial AgNPs standard suspensions used for size calibration.

Size Reported in Product Certificate ^a^			DLS Analysis Value ^b^ (nm)		Relative Deviation ^c^ (%)
Nominal Size (nm)	TEM Diameter (nm)	Hydrodynamic Diameter (nm)	Z-Average (nm)	PDI	
20	20 ± 3	25	26.5 ± 0.6	0.244	6.0
40	41 ± 5	44	42.8 ± 0.0	0.132	2.7
60	59 ± 5	60	60.7 ± 0.6	0.134	1.2
80	81 ± 8	81	80.7 ± 1.0	0.077	0.9
100	97 ± 13	101	101.3 ± 1.0	0.086	0.3

^a^ Size information as reported in the manufacturer’s certificates. ^b^ Z-average and polydispersity index (PDI) obtained by DLS measurement (n = 3). ^c^ Relative deviation obtained by comparison of the Z-average measured by DLS with the hydrodynamic size declared in the manufacturer’s certificate.

**Table 2:** Evaluation of accuracy and repeatability in measurement of concentration and size of AgNPs in milk by AF4-ICP-MS and SP-ICP-MS.

		AF4-ICP-MS					SP-ICP-MS				
Accuracy (%) ^a^	Concentration ^b^	20 µg/L	40 µg/L	60 µg/L	80 µg/L	100 µg/L	2 µg/L	4 µg/L	6 µg/L	8 µg/L	10 µg/L
		96 ± 2	101 ± 3	99 ± 2	100 ± 0	100 ± 1	100 ± 4	101 ± 1	100 ± 2	99 ± 1	101 ± 1
	Size ^c^	20 nm	40 nm	60 nm	80 nm	100 nm	20 nm	40 nm	60 nm	80 nm	100 nm
		87 ± 0	106 ± 1	103 ± 1	101 ± 0	98 ± 0	97 ± 4	103 ± 3	99 ± 1	100 ± 0	100 ± 0
Repeatability (RSD, %) ^a^	Concentration ^b,c^	2 ± 1					2 ± 1				
	Size ^b,d^	1 ± 1					1 ± 1				

^a^ Results are presented as mean ± standard deviation (n = 3). ^b^ 60 nm AgNPs standard suspensions were selected as representative example at various concentrations. ^c^ AgNPs standard suspensions were spiked at 60 µg/mL (for AF4-ICP-MS analysis) or 6 µg/L (for SP-ICP-MS analysis), respectively, as representative examples. ^d^ AgNPs standard suspensions were spiked at 285 µg/L for AF4-ICPMS and SP-ICPMS analysis.

**Table 3:** Detection and quantification limits of AgNPs in milk using AF4-ICP-MS and SP-ICP-MS.

Limit of Detection (µg/L)		Limit of Quantification (µg/L)	
AF4-ICP-MS	SP-ICP-MS	AF4-ICP-MS	SP-ICP-MS
3.2	0.5	10.6	5.6

**Table 4:** Determination of total silver content from breast milk storage bags under various scenarios as well as summary of incubation conditions for migration studies in this work.

Sample	Total Silver Content by ICP-MS (n = 3)	
	Microwave-Assisted Digestion (mg/kg)	Migration into Food Simulant of 10% Ethanol (µg/L)
Breast milk storage bag 1	3.48 ± 0.32	0.16 ± 0.01
Breast milk storage bag 2	15.79 ± 0.67	0.28 ± 0.01
Breast milk storage bag 3	22.89 ± 0.66	0.23 ± 0.01
Different incubation conditions for migration studies in this work		
S/N	Migration test	Migration condition
1	Simulant: 10% ethanol	40 °C for 10 days
2	Milk	37 °C for 1 h, 4 °C for 5 days and −20 °C for 10 days
3	Milk (harsh condition)	(1) Heat at 70 °C for 1 h
		(2) Boil at 100 °C for ½ h
		(3) Microwave for 1 min

## Data Availability

Not applicable.

## Sample Availability

Not available.

## References

[1] Christensen F.M., Johnston H.J., Stone V., Aitken R.J., Hankin S., Peters S., Aschberger K. (2010). Nano-silver–feasibility and challenges for human health risk assessment based on open literature. Nanotoxicology.

[2] Nowack B., Krug H.F., Height M. (2011). 120 years of nanosilver history: Implications for policy makers. Environ. Sci. Technol..

[3] Williams K.M., Gokulan K., Cerniglia C.E., Khare S. (2016). Size and dose dependent effects of silver nanoparticle exposure on intestinal permeability in an in vitro model of the human gut epithelium. J. Nanobiotechnol..

[4] Faunce T., Bruce A., Donohoo A., Hull M., Bowman D. (2014). Nanomaterial governance, planetary health, and the sustainocene transition. Nanotechnology Environmental Health and Safety.

[5] Carbone M., Donia D.T., Sabbatella G., Antiochia R. (2016). Silver nanoparticles in polymeric matrices for fresh food packaging. J. King Saud Univ.-Sci..

[6] (2011). Regulation (EU) No 1169/2011 of the European Parliament and of the Council on the Provision of Food Information to Consumers.

[7] EFSA Panel on Food Contact Materials, Enzymes, Flavourings and Processing Aids (CEF) (2011). Scientific Opinion on the safety evaluation of the substance, silver zeolite A (silver zinc sodium ammonium alumino silicate), silver content 2–5%, for use in food contact materials. EFSA J..

[8] EFSA Panel on Food Contact Materials, Enzymes and Processing Aids (CEP) (2021). Safety assessment of the substance silver nanoparticles for use in food contact materials. EFSA J..

[9] Mackevica A., Olsson M.E., Hansen S.F. (2016). Silver nanoparticle release from commercially available plastic food containers into food simulants. J. Nanopart Res..

[10] Mackevica A., Olsson M.E., Hansen S.F. (2017). The release of silver nanoparticles from commercial toothbrushes. J. Hazard. Mater..

[11] Hetzer B., Burcza A., Graf V., Walz E., Greiner R. (2017). Online-coupling of AF4 and single particle-ICP-MS as an analytical approach for the selective detection of nanosilver release from model food packaging films into food simulants. Food Control.

[12] Ding R., Yang P., Yang Y., Yang Z., Luo L., Li H., Wang Q. (2018). Characterisation of silver release from nanoparticle-treated baby products. Food Addit. Contam. Part A.

[13] Metak A.M., Nabhani F., Connolly S.N. (2015). Migration of engineered nanoparticles from packaging into food products. LWT-Food Sci. Technol..

[14] Deng J., Ding Q.M., Li W., Wang J.H., Liu D.M., Zeng X.X., Liu X.Y., Ma L., Deng Y., Wei S. (2020). Preparation of Nano-Silver-Containing Polyethylene Composite Film and Ag Ion Migration into Food-Simulants. J. Nanosci. Nanotechnol..

[15] Yang Y., Zhang X., Li H., Wang H., Liu N., Qiu S., Bi H. (2021). Study on the Migration of Silver Nanoparticles from Nano silver Food Packages into Food Liquid. Sch. J. Agric. Vet. Sci..

[16] Corps Ricardo A.I., García S.A., Bernardo F.J.G., Ríos Á., Rodríguez Martín-Doimeadios R.C. (2021). Rapid assessment of silver nanoparticle migration from food containers into food simulants using a qualitative method. Food Chem..

[17] Labbok M.H., Clark D., Goldman A.S. (2004). Breastfeeding: Maintaining an irreplaceable immunological resource. Nat. Rev. Immunol..

[18] Singh S.P., Bhargava C.S., Dubey V., Mishra A., Singh Y. (2017). Silver nanoparticles: Biomedical applications, toxicity, and safety issues. Int. J. Pharm. Pharm. Sci..

[19] Hardy A., Benford D., Halldorsson T., Jeger M.J., Knutsen H.K., More S., Naegeli H., Noteborn H., Ockleford C., EFSA Scientific Committee (2017). Guidance on the risk assessment of substances present in food intended for infants below 16 weeks of age. EFSA J..

[20] Ramos K., Ramos L., Camara C., Gomez-Gomez M.M. (2014). Characterization and quantitation of silver nanoparticles in nutraceuticals and beverages by asymmetric flow field flow fractionation coupled with inductively coupled plasma mass spectrometry. J. Chromatogr. A.

[21] Loeschner K., Navratilova J., Grombe R., Linsinger T.P.J., Kobler C., Molhave K., Larsen E.H. (2015). In-house validation of a method for determination of silver nanoparticles in chicken meat based on asymmetric flow field-flow fractionation and inductively coupled plasma mass spectrometric detection. Food Chem..

[22] Artiaga G., Ramos K., Ramos L., Camara C., Gomez-Gomez M. (2015). Migration and characterisation of nanosilver from food containers by AF4-ICP-MS. Food Chem..

[23] Peters R.J.B., Rivera Z.H., Bemmel G.V., Marvin H.J.P., Weifel S., Bouwmeester H. (2014). Development and validation of single particles ICP-MS for sizing and quantitative determination of nano-silver in chicken meat. Anal. Bioanal. Chem..

[24] Witzler M., Kullmer F., Hirtz A., Gunther K. (2016). Validation of gold and silver nanoparticles analysis in fruit juice by single-particles ICP-MS without sample pretreatment. J. Agric. Food Chem..

[25] Aznar R., Barahona F., Geiss O., Ponti J., José Luis T., Barrero-Moreno J. (2017). Quantification and size characterisation of silver nanoparticles in environmental aqueous samples and consumer products by single particle-ICPMS. Talanta.

[26] Grasso A., Ferrante M., Arena G., Salemi R., Zuccarello P., Fiore M., Copat C. (2021). Chemical Characterization and Quantification of Silver Nanoparticles (Ag-NPs) and Dissolved Ag in Seafood by Single Particle ICP-MS: Assessment of Dietary Exposure. Int. J. Environ. Res. Public Health.

[27] Jokar M., Pedersen G.A., Loeschner K. (2017). Six open questions about the migration of engineered nano-objects from polymer-based food-contact materials: A review. Food Addit. Contam. Part A.

[28] Geiss O., Cascio C., Gilliland D., Franchini F., Barrero-Moreno J. (2013). Size and mass determination of silver nanoparticles in an aqueous matrix using asymmetric field flow fractionation coupled to inductively coupled plasma mass spectrometer and ultraviolet–visible detectors. J. Chromatogr. A.

[29] Bocca B., Battistini B., Petrucci F. (2020). Silver and gold nanoparticles characterization by SP-ICP-MS and AF4-FFF-MALS-UV-ICP-MS in human samples used for biomonitoring. Talanta.

[30] Taboada-López M.V., Bartczak D., Cuello-Núñez S., Goenaga-Infante H., Bermejo-Barrera P., Moreda-Piñeiro A. (2021). AF4-UV-ICP-MS for detection and quantification of silver nanoparticles in seafood after enzymatic hydrolysis. Talanta.

[31] Ricardo A.I.C., Fariñas N.R., Bernardo F.J.G., Martín-Doimeadios R.C.R., Ríos Á. (2021). Screening-confirmation strategy for nanomaterials involving spectroscopic analytical techniques and its application to the control of silver nanoparticles in pastry samples. Spectrochim. Acta Part A Mol. Biomol. Spectrosc..

[32] Weigel S., Peters R., Loeschner K., Grombe R., Linsinger T.P.J. (2017). Results of an interlaboratory method performance study for the size determination and quantitation of silver nanoparticles in chicken meat by single-particles inductively coupled plasma mass spectrometry (sp-ICP-MS). Anal. Bioanal. Chem..

[33] Mitrano D.M., Barber A., Bednar A., Westerhoff P., Higgins C.P., Ranville J.F. (2012). Silver nanoparticle characterization using single particle ICP-MS (SP-ICP-MS) and asymmetrical flow field flow fractionation ICP-MS (AF4-ICP-MS). J. Anal. At. Spectrom..

[34] Marioli M., Kok W.T. (2020). Continuous asymmetrical flow field-flow fractionation for the purification of proteins and nanoparticles. Sep. Purif. Technol..

[35] Loeschner K., Navratilova J., Kobler C., Molhave K., Wagner S., von der Kammer F., Larsen E.H. (2013). Detection and characterization of silver nanoparticles in chicken meat by asymmetric flow field fractionation with detection by conventional or single particle ICP-MS. Anal. Bioanal. Chem..

[36] Correia M., Loeschner K. (2018). Detection of nanoplastics in food by asymmetric flow field-flow fractionation coupled to multi-angle light scattering: Possibilities, challenges and analytical limitation. Anal. Bioanal. Chem..

[37] Li B., Chua S.L., Ch’ng A.L., Yu D., Koh S.P., Phang H., Chiew P. (2020). An effective approach for size characterization and mass quantification of silica nanoparticles in coffee creamer by AF4-ICP-MS. Anal. Bioanal. Chem..

[38] Makan A.C., Spallek M.J., du Toit M., Klein T., Pasch H. (2016). Advanced analysis of polymer emulsions: Particle size and particle size distribution by field-flow fractionation and dynamic light scattering. J. Chromatogr. A.

[39] Bolea E., Jiménez-Lamana J., Laborda F., Castillo J.R. (2011). Size characterization and quantification of silver nanoparticles by asymmetric flow field-flow fractionation coupled with inductively coupled plasma mass spectrometry. Anal. Bioanal. Chem..

[40] Poda A.R., Bednar A.J., Kennedy A.J., Harmon A., Hull M., Mitrano D.M., Ranville J.F., Steevens J. (2011). Characterization of silver nanoparticles using flow-field flow fractionation interfaced to inductively coupled plasma mass spectrometry. J. Chromatogr. A.

[41] Bednar A.J., Poda A.R., Mitrano D.M., Kennedy A.J., Gray E.P., Ranville J.F., Hayes C.A., Crocker F.H., Steevens J.A. (2013). Comparison of on-line detectors for field flow fractionation analysis of nanomaterials. Talanta.

[42] Choi J.I., Chae S.J., Kim J.M., Choi J.C., Park S.J., Choi H.J., Bae H., Park H.J. (2018). Potential silver nanoparticles migration from commercially available polymeric baby products into food simulants. Food Addit. Contam. Part A.

[43] European Commission (2011). Commission Regulation (EU) No 10/2011 of 14 January 2011 on Plastic Materials and Articles Intended to Come into Contact with Food.

[44] Ntim S.A., Thomas T.A., Begley T.H., Noonan G.O. (2015). Characterization and potential migration of silver nanoparticles from commercially available polymeric food contact material. Food Addit. Contam. Part A.

[45] Loeschner K., Brabrand M.S.J., Sloth J.J., Larsen E.H. (2014). Use of alkaline or enzymatic sample pretreatment prior to characterization of gold nanoparticles in animal tissue by single-particles ICPMS. Anal. Bioanal. Chem..

[46] Peters R., Herrera-Rivera Z., Undas A., van der Lee M., Marvin M., Bouwmeester H., Weigel S. (2015). Single particles ICP-MS combined with a data evaluation tool as a routine technique for the analysis of nanoparticles in complex matrices. J. Anal. At. Spectrom..

